# The Effect of Tissue Inhibitor of Metalloproteinases on Scar Formation after Spinal Cord Injury

**DOI:** 10.3390/cells13181547

**Published:** 2024-09-14

**Authors:** Raveena R. Mishra, Brooke E. Nielsen, Melissa A. Trudrung, Samuel Lee, Luke J. Bolstad, Daniel J. Hellenbrand, Amgad S. Hanna

**Affiliations:** 1 Department of Neurosurgery, School of Medicine and Public Health, University of Wisconsin-Madison, Madison, WI 53706, USA; rrmishra@wisc.edu (R.R.M.); benielsen@wisc.edu (B.E.N.); trudrung@wisc.edu (M.A.T.); slee2335@wisc.edu (S.L.); ljbolstad@wisc.edu (L.J.B.); 2 Department of Biomedical Engineering, University of Wisconsin-Madison, Madison, WI 53706, USA

**Keywords:** TIMP-1, spinal cord injury, astrocytes, activation, blood–brain barrier, blood–spinal cord barrier

## Abstract

Spinal cord injury (SCI) often results in permanent loss of motor and sensory function. After SCI, the blood–spinal cord barrier (BSCB) is disrupted, causing the infiltration of neutrophils and macrophages, which secrete several kinds of cytokines, as well as matrix metalloproteinases (MMPs). MMPs are proteases capable of degrading various extracellular matrix (ECM) proteins, as well as many non-matrix substrates. The tissue inhibitor of MMPs (TIMP)-1 is significantly upregulated post-SCI and operates via MMP-dependent and MMP-independent pathways. Through the MMP-dependent pathway, TIMP-1 directly reduces inflammation and destruction of the ECM by binding and blocking the catalytic domains of MMPs. Thus, TIMP-1 helps preserve the BSCB and reduces immune cell infiltration. The MMP-independent pathway involves TIMP-1’s cytokine-like functions, in which it binds specific TIMP surface receptors. Through receptor binding, TIMP-1 can stimulate the proliferation of several types of cells, including keratinocytes, aortic smooth muscle cells, skin epithelial cells, corneal epithelial cells, and astrocytes. TIMP-1 induces astrocyte proliferation, modulates microglia activation, and increases myelination and neurite extension in the central nervous system (CNS). In addition, TIMP-1 also regulates apoptosis and promotes cell survival through direct signaling. This review provides a comprehensive assessment of TIMP-1, specifically regarding its contribution to inflammation, ECM remodeling, and scar formation after SCI.

## 1. Introduction

The National Spinal Cord Injury Statistical Center reported that, in 2017, 285,000 people were living with a spinal cord injury (SCI) in the United States, with an incidence rate of about 17,500 per year [1]. Most SCIs result in varying levels of motor, sensory, and visceral dysfunction below the lesion level [2]. As a result, people living with SCI experience significant lifestyle changes, including loss of mobility and independence, which reduce their quality of life [3]. Although several neuroprotective strategies show promising results in improving functional recovery in animal models after SCI, currently, no treatments significantly improve function in humans after SCI, except for surgical decompressions [4].

The initial mechanical damage after SCI disrupts the blood–spinal cord barrier (BSCB) and causes cell death [2,3,5,6,7]. The breakdown in BSCB and the damage-associated molecular patterns released from lysed cells activate astrocytes and microglia to release cytokines, which amplify inflammation after a SCI [8]. Damage to the BSCB allows leukocytes, including neutrophils and macrophages, from the periphery to be guided to the lesion [2,4]. Leukocytes secrete matrix metalloproteinases (MMPs) that dramatically augment inflammation by engaging in the destruction of the extracellular matrix (ECM) [7,9]. The release of MMPs disrupts cell growth and remodeling after injury and leads to destruction of the myelin sheath [10]. In addition, when the cells’ adhesion to the ECM is disrupted, the cells undergo apoptosis [11]. Astrocytes are directed towards the site of injury and proliferate rapidly, forming a glial scar that protects the remaining intact spinal cord [2]. However, the glial scar inhibits axonal regeneration in the chronically injured spinal cord [2,5,7,12,13,14].

The inhibition of MMPs results in the preservation of the BSCB and reduced neutrophil migration to the site [15]. Like other tissue inhibitor matrix metalloproteinases (TIMPs), TIMP-1 functions as a competitive inhibitor of MMPs, but TIMP-1 also possesses cytokine-like properties. Under homeostatic conditions, TIMP-1 is at a low concentration within the central nervous system (CNS); several studies show that TIMP-1 is significantly upregulated post-SCI [16,17,18]. This review discusses the role TIMP-1 plays after SCI, including its direct effect on inhibiting MMPs, effects through cell receptor binding, and indirect effects caused by reducing the ECM turnover.

## 2. Discussion

For this review, we searched PubMed to find articles addressing the role of TIMP-1 in SCI using the following keywords: (TIMP-1), (spinal cord injury), (astrocytes), (activation), (blood–brain barrier), and (blood–spinal cord barrier). This review was limited to only TIMP-1. TIMP-2, TIMP-3, and TIMP-4 were all excluded from the review due to their lack of upregulation post-SCI [18]. The studies were also not based on animal models. In addition, TIMP-1’s association with the peripheral nervous system was excluded. Studies involving the brain or another focus were only included if reasonable extrapolations to the spinal cord (SC) could be made.

### 2.1. TIMP-1 Structure and Background

The inhibitory effect of TIMP-1 was discovered in the 1970s, when it was initially characterized as a collagenase inhibitor [19,20]. Later, it was found that the molecule also inhibits the activity of gelatinases (MMP-2 and MMP-9) and a proteoglycanase (MMP-3), leading to its renaming as TIMP [21]. TIMPs are a family of four proteins (TIMP-1, TIMP-2, TIMP-3, and TIMP-4) that act as inhibitors of metalloproteinases to balance MMP functions [19]. Humans have 23 MMPs that are inhibited by the cumulative activities of all four TIMPs [22]. The MMP inhibitory activity of TIMPs is a function of their wedge-shaped N-terminal domain, which is formed by disulfide bonds [23,24,25]. This wedge slots into the catalytic site of MMPs, occupying 75% of the site, allowing TIMP-1 to inhibit MMP enzymatic activity. When TIMP-1 interacts with a MMP, the zinc ion located in the MMP catalytic site is bidentately chelated by the amino group of the N-terminal and the carbonyl group of Cys1 of TIMP. Chelation displaces the water molecule bound to the zinc ion and inhibits the MMP [26].

The wide variety of TIMPs present in Gnathostomes arose from multiple whole gene duplications (WGDs) over the course of evolution, which allowed each copy of the TIMP gene to acquire new mutations, giving way to each of their specialized abilities [19,27,28,29,30,31,32]. While TIMPs are present in the Gnathostomes superclass, TIMP-1 is absent in a couple of classes, such as the Aves and Teleostei fish [27,28]. The lack of TIMP-1 in Aves is thought to have happened due to the reduction in genome size associated with their adaptation to flight. In contrast, the lack of TIMP-1 in Teleostei fish is believed to be the outcome of the inactivation of redundant genes following a WGD event [27,28,33,34,35]. TIMP-1’s absence in Teleostei fish, such as zebrafish, stimulates some interesting hypotheses of TIMP-1’s potential involvement in inhibiting the process of neural regeneration, as zebrafish can regenerate nervous tissue following an injury. However, studies that have explored this phenomenon have focused on proteins outside of TIMP-1’s direct interactions, such as CTGFa, Cav1, ANP32a, BDNF, FGF, and sonic hedgehog [36,37,38,39,40,41,42,43,44,45,46,47,48,49,50]. One study did look into MMP-9’s effect on neural regeneration, but this was outside the scope of SCIs and focused on the regeneration of photoreceptors [51].

### 2.2. TIMP-1 Directly Inhibits MMP Activity

All MMPs are translated as inactive zymogens (proMMPs) with an auto-inhibitory propeptide domain located at the N-terminal that must be removed to activate the protease. Membrane-type MMPs and some secreted MMPs undergo activation during their transit through the secretory pathway via the action of pro-protein convertases such as furin, but most secreted MMPs are activated in the extracellular environment through the actions of other extracellular proteases [52]. This web of cross-activating proteases remains poorly studied in vivo but plays a central role in inflammatory pathologies like SCIs.

**Figure 1:**
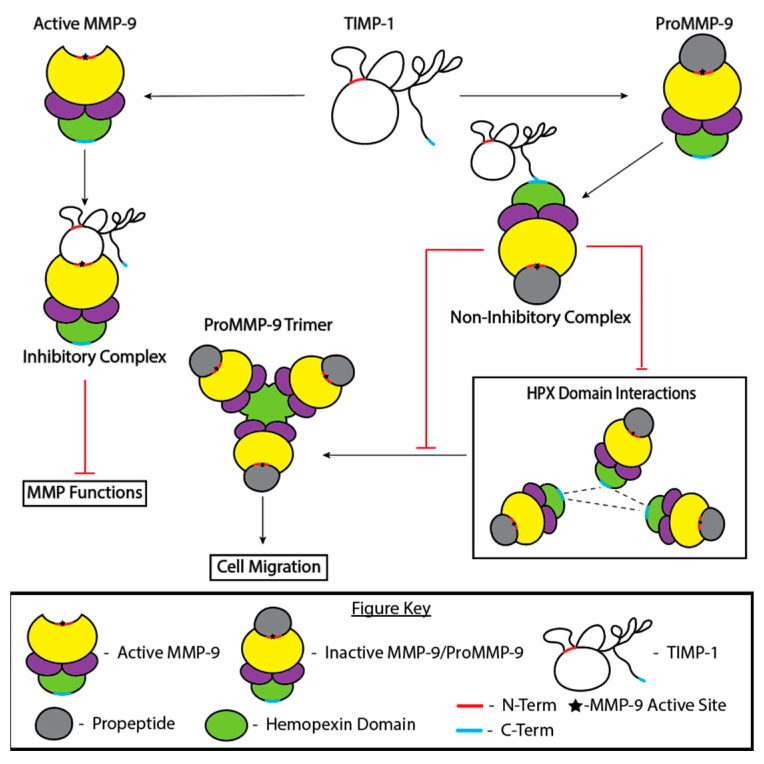
MMP–TIMP interactions. TIMP-1 binds active MMP-9 to form the inhibitory complex, which blocks the active site of MMP-9 and inhibits the enzymatic activity of the metalloproteinase (left pathway). TIMP-1 also binds to proMMP-9, the form of MMP-9 that is secreted and is inactive until the pro-domain is cleaved [54]. The formation of the non-inhibitory complex blocks the interactions between the hemopexin domains of proMMP-9, which blocks the formation of proMMP-9 homotrimers, and has implications in cell migration [55,56,57,58].

Upon the cleavage of MMP-9’s propeptide from the catalytic site, the molecule transitions from inactive proMMP-9 to active MMP-9 [53]. TIMP-1 can act upon both forms of MMP-9, one of which is through the formation of an inhibitory complex between the N-terminal catalytic site of active MMP-9 and the wedge-shaped loop located at the N-terminal of the TIMP-1 (Figure 1). This complex inhibits MMP-9’s catalytic activity. TIMP-1 can also form a non-inhibitory complex by binding to the C-terminal hemopexin-like domain of proMMP-9, which blocks the cleavage of the propeptide, halting MMP activity [54] (Figure 1). The hemopexin domain is responsible for the formation of proMMP-9 complexes, later to be classified as homotrimers [55,56]. These proMMP-9 homotrimers initiate communication between CD44 and EGFR to commence the activation of downstream effectors to facilitate cell migration in epithelial cells and other cell types [57]. In the presence of TIMP-1, the hemopexin domains of proMMP-9 cannot interact, blocking trimer formation and leading to decreased migration in epithelial cells [58] (Figure 1). Each pathway that TIMP-1 acts upon has been identified, but the degree to which each pathway occurs needs further exploration, as the ratio of proMMP-9 to MMP-9 is still unknown [59]. However, new approaches to investigate the post-translational regulation of MMP activation in vivo now exist. Epitope-mediated MMP activation (EMMA) is a technique that marks the propeptide and C-terminal domains of MMPs with epitopes to allow researchers to distinguish between the presence of proteolytically processed MMPs or inactive forms of MMPs [60]. The utilization of this approach would be a profitable avenue for future investigations of MMP-9 activation.

**Figure 2:**
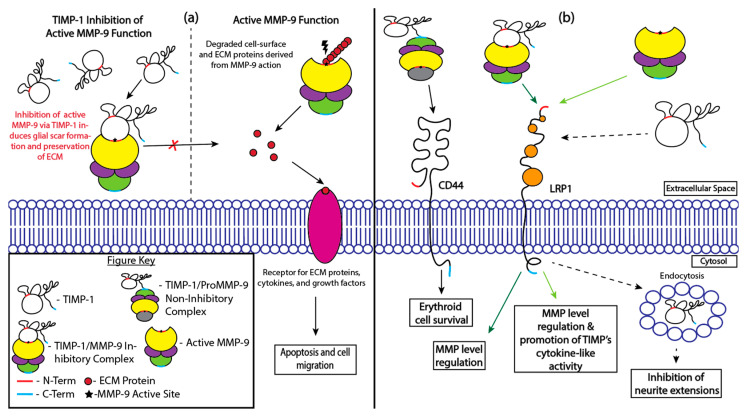
TIMP-1 actions involving MMPs. (**a**) Direct inhibition of MMP-9 function by TIMP-1. TIMP-1 binds free MMPs, which decreases ECM turnover [13,62]. Thus, because there are less degraded materials in the presence of TIMP-1, apoptosis and cell migration are modulated [10,25,61]. (**b**) Additional MMP-dependent actions of TIMP-1. The inhibitory and non-inhibitory complexes formed by the two molecules can further bind cell surface receptors CD44 and LRP1 for the purposes of cell regulation and cell survival [63,64]. When the two molecules are not in their complex state, they still process the function to bind to LRP1, having implications in either promoting TIMP cytokine-like activity or inhibiting neurite extensions [64,65].

MMPs engage in the destruction of the ECM and regulate cells’ interactions with the ECM (Figure 2a) [25]. This influences normal cell processes, such as tissue remodeling and growth [61]. MMPs have roles in BSCB destruction, as there is an augmented expression of MMP-9 post-SCI. Although MMP-9 levels increase post-SCI and peak 24 h post-injury, MMP-9 is undetectable 7 days post-injury in mice models [15]. After SCIs, TIMP-1 is also significantly increased in rat and mice models, with its earliest augmentation occurring 6 h post-injury and remaining significantly higher than the baseline values for over a week post-injury [15,17,18].

Because TIMP-1 inhibits the functions of MMPs, studies knocking out MMPs have speculated about the effects of TIMP-1 on the ECM turnover. When MMP-9 was knocked out in mice that endured a SCI, there was more preservation of the BSCB, less migration of neutrophils to the injury site, and more functional recovery post-injury [15]. Similarly, when MMP-12 was knocked out in mice, functional recovery increased [66].

Both TIMP-1/MMP inhibitory and non-inhibitory complexes bind to various cell surface receptors to either promote cell survival or regulate cell behavior (Figure 2b). When the non-inhibitory TIMP-1/proMMP-9 complex binds receptor CD44, there is an increased survival of red blood cells [63]. When active MMP-9, both free or bound to TIMP-1, binds to low-density lipoprotein receptor-related protein-1 (LRP-1), regulation of the MMP-9 levels occurs [64]. Additionally, the binding of free MMP-9 to LRP-1 allows for TIMP-1’s cytokine-like activity to be favored. However, free TIMP-1 binding to LRP-1 results in the internalization of TIMP-1 and decreases neurite outgrowth [65].

A Disintegrin And Metalloproteinase (ADAM) and A Disintegrin And Metalloproteinase ThromboSpondin motifs (ADAMTS) are other zinc-dependent metalloproteinases that are in the same class of proteins as MMPs. These three proteins share similar functions, as they are responsible for tissue remodeling through the degradation of the ECM. What distinguishes ADAM and ADAMTS from MMPs is the presence of a disintegrin domain that takes the place of the hemopexin-like domain of the MMP [67]. Most ADAMs contain a transmembrane domain, as well as a cytoplasmic domain, allowing these proteins to be localized at the cell surface. However, ADAMTS lack these domains, making them a secretory protein that is found within the ECM [68].

There have been 21 members of the ADAM and 19 members of ADAMTS reported in the human genome [69,70]. The most notable members of this family in the context of SCIs are ADAM-17 and ADAM-10. ADAM-17 plays a pivotal role in the recovery process after a SCI, as it promotes the survival of microglia though the neuroprotective EGFR/MAPK pathway [71]. The survival of these cells is vital for the recovery process post-SCI, as they coordinate the injury response in a damaged CNS [72]. However, TIMP-3 inhibits ADAM 17, and the upregulation of this inhibitor would have detrimental effects on the recovery process [73]. TIMP-3 and TIMP-1 also inhibit ADAM-10, which is proven to be useful in the context of SCIs due to ADAM-10’s ability to break down myelin basic protein (MBP), a protein that supports axonal regeneration post-SCI [74,75]. These findings highlight TIMP-1’s unique role in improving recovery after SCIs compared to TIMP-3. This leads to the speculation that TIMP-1’s presence post-SCI supports axonal regrowth by inhibiting ADAM-10 and allows ADAM-17 to remain active to promote microglial survival, though future investigations are necessary to solidify this claim.

### 2.3. TIMP-1’s Direct Effect through Receptor Binding

**Figure 3:**
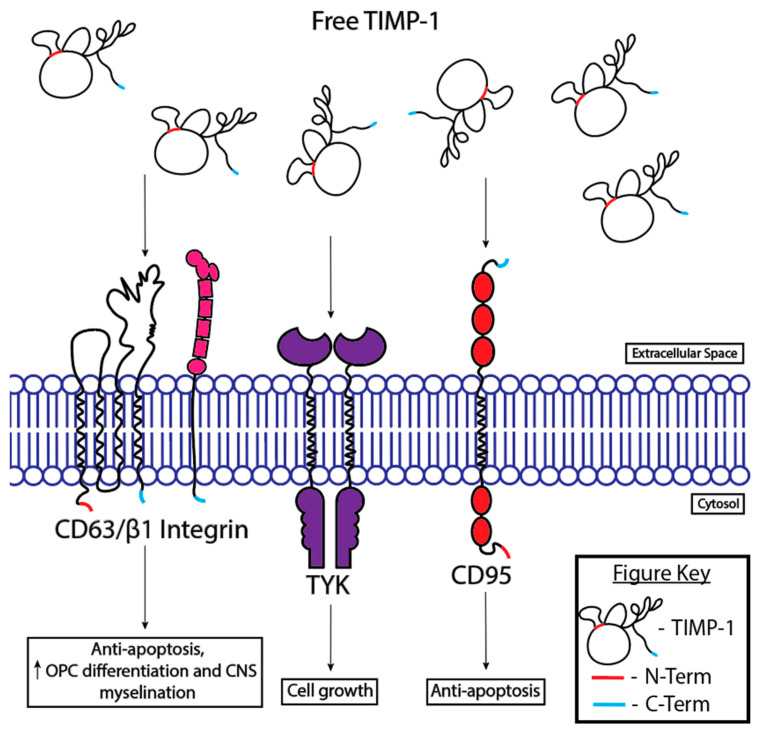
Cytokine-like functions of TIMP-1. TIMP-1-binding CD63/beta1 integrin, TYK, or CD95 can all have CNS preservation/growth effects [79,80,81,82].

TIMP-1 also contributes to the repair mechanism within the CNS through MMP-independent actions. In this manner, TIMP-1 mediates inflammation by blocking the dissociation of cytokines on the surface of the cell [30]. TIMPs also have cytokine-like functions, binding specific TIMP surface receptors (Figure 3) [9].

Initially, TIMP-1 was identified from cDNA encoding for a protein with erythroid potentiating activity, with the supplementation of TIMP-1 promoting erythroid precursor cell growth through direct cell surface binding [76]. TIMP-1’s activity in this context is likely mediated by autocrine or paracrine signaling, as even low levels of anti-TIMP-1 monoclonal antibodies (clone-76Cl) in cell culture media abolished TIMP-1’s growth-promoting effect [77]. The cell growth activity of TIMP-1 through cell surface signaling remains prominent even when its MMP inhibitory activity ceases [77,78]. Free TIMP-1 is required for this activity; once TIMP-1 binds MMPs, it cannot proceed with MMP-independent growth-promoting activity [77].

TIMP-1 stimulates the growth of several cell types, including keratinocytes [83], aortic smooth muscle cells, skin epithelial cells [77], corneal epithelial cells [84], malignant cancer cells [81,82], and astrocytes [16,85]. The molecule also prevents scar formation by limiting MMP-activated transforming growth factor-β (TGF-β) release, which is responsible for augmenting ECM accumulation or fibrosis [30]. On cell surfaces, TIMP-1 reduces inflammation by preventing the removal of certain cell surface cytokines and cytokine receptors. This indirectly affects the ECM turnover by dampening the inflammatory response. Therefore, TIMP-1 can decrease inflammation and reduce ECM accumulation/scar formation, depending on the metalloproteinase profile present in the site [86,87].

TIMP-1 also plays a role in activating Ras, which stimulates cell growth. TIMP-1 activates Ras through a tyrosine kinase (TYK)-mediated pathway, further activating Raf-1, a proto-oncogene, as part of the mitogen-activated protein kinase (MAPK) pathway (Figure 3) [81]. Additionally, TIMP-1 regulates interleukin (IL)-10 in germinal centers of B cells, promoting cell survival [88]. The presence of TIMP-1 also suppresses apoptosis in malignant cancer cell lines, potentially through the CD95 pathway, thereby blocking cell death (Figure 3) [9,82]. Thus, TIMP-1’s antiapoptotic activity is independent of MMP inhibition [82].

TIMP-1 can also suppress apoptosis through binding the receptor CD63 (Figure 3) [79]. TIMP-1 signaling is mediated through CD63/β1-integrin/Akt [79,80,89]. The binding of TIMP-1 to the CD63 receptor assists in the differentiation and trophic activity of oligodendrocytes. CD63, β1-integrin, and TIMP-1 activate Akt to phosphorylate GSK-3β, leading to an increase in the activity of β-catenin, which promotes the differentiation of oligodendrocyte progenitor cells (OPCs) and CNS myelination [80]. However, proMMP-9 competes with CD63 to bind to the N-terminus of TIMP-1, with proMMP-9 having a higher binding affinity to TIMP-1 than CD63. The binding of proMMP-9 to TIMP-1 inhibits TIMP-1’s antiapoptotic effect, which can have implications on disease treatment [9]. It is important to note that active MMP-9 may also compete with proMMP-9 to bind to TIMP-1 to inhibit binding to CD63, as active MMP-9 has two open sites, the active site and the hemopexin region, whereas proMMP-9 only has one, the hemopexin region. No studies have elucidated the difference in the binding affinity of TIMP-1 to active or proMMP-9. Thus, future studies should explore this topic to clarify which form of MMP-9 has a greater role in inhibiting the binding of TIMP-1 to CD63.

It should be noted that the expression of local inflammatory molecules—specifically, IL-1β, IL-6, TNF-α, and IL-10—do not appear to be directly augmented when TIMP-1 is decreased. TIMP-1’s pain-reducing and inflammation-reducing effects are due to its MMP-dependent or receptor-binding pathways rather than directly altering the local cytokine microenvironment [90].

### 2.4. Timeline of Upregulation, Pathways, and Effects

As mentioned before, MMP-9 and TIMP-1 are both upregulated following the event of a SCI. However, the duration and the species present during their upregulation varies between the two molecules, leading to varying signaling pathways chosen over the course of time. In a study by Noble et al., mice that underwent a SCI had different species of MMP-9 present as the days to follow after the injury progressed. At 24 h post-injury, the MMP-9/TIMP complex, proMMP-9, and active MMP-9 were present in the tissue harvested, whereas, at 72 h and into a week post-injury, the only prominent species present was proMMP-9 [15]. In a study by Hellenbrand et al., rats that underwent a SCI exhibited elevated levels of TIMP-1 that peaked at 24 h and remained significantly upregulated for more than a week after injury [17].

It can be hypothesized that TIMP-1’s primary focus at the 24-h time point is to dampen MMP-9’s effects at the site of injury. TIMP-1 can do this through multiple different modes. One is by directly inhibiting MMP-9 catalytic activity, which reduces the ECM turnover, enables cell survival, inhibits leukocyte migration, and promotes the formation of a glial scar [10,13,25,61,62]. The other mode in which TIMP-1 dampens MMP-9’s effect at the injury site is through the TIMP-1/MMP-9 complex binding to LRP-1. The binding of the inhibitory complex to LRP-1 activates MMP-9-level regulation, decreasing the presence of MMP-9. Free MMP-9 present during the 24-h phase also binds to LRP-1, further regulating the MMP-9 levels [64]. LRP-1 may be one of the main contributors to decreasing MMP-9 levels in the timeframe of 24 h to 72 h after injury, while TIMP-1 works to inhibit the active metalloproteinase function.

ProMMP-9 is present over the course of the 72 h after injury as well, which has implications on the formation of trimer complexes that enhance cell migration [55,56,57]. It can be assumed that TIMP-1’s presence would work to bind to proMMP-9 to block the formation of these trimers and decrease leukocyte migration towards the injury site in this timeframe [58]. The presence of a TIMP-1/proMMP-9 complex would affect erythroid cells, as the binding of this complex to CD44 promotes the survival of these cells [63].

Since active MMP-9 levels decrease after 24 h after injury, TIMP-1’s cytokine-like activity is most likely favored past this time point. It would be expected to see TIMP-1 binding to the CD63/β1 integrin complex to prevent apoptosis and promote OPC differentiation and CNS myelination, as well as aid in maintaining the integrity of the BSCB [79,80,91]. Furthermore, TIMP-1 would be expected to bind to TYK and CD95 to further prevent apoptosis and promote cell growth [81,82]. The only drawback in free TIMP-1 present in this timeframe is due to the binding of the molecule to LRP-1, which inhibits neurite extensions [65]. Future investigations may want to investigate engineering a molecule to introduce after a SCI to block the binding of TIMP-1 and LRP-1 in order to promote neurite extension. It should also be noted that the aforementioned claims of TIMP-1’s primary role at each time point are hypotheticals made from the culmination of the results of rat and mice studies. Research that monitors what pathways are activated at each time point needs to be performed in one of these organisms to solidify these claims.

### 2.5. The Effect of TIMP-1 in the CNS

Overall, TIMP-1 has a multitude of effects in the CNS, and its pathways are extremely complex due to the wide variety of cells it acts upon and its presence varying throughout the timeline of the inflammatory process. TIMP-1 inhibits MMPs (which have downstream anti-inflammatory effects), induces tissue remodeling, and inhibits angiogenesis [92,93,94]. Within the CNS, TIMP-1 directly and indirectly affects various cells such as astrocytes, microglia, macrophages, neurons, and neuronal stem cells. When acting on these cells, TIMP-1 can further influence the neuroregeneration and neuroprotective processes.

#### 2.5.1. TIMP-1’s Effect on Astrocytes

One of TIMP-1’s fundamental functions is the activation of astrocytes. Moore et al. demonstrated a loss of function of TIMP-1 in post-lesion mice that coincided with a dramatic reduction in the number of astrocytes in the developing CNS [16]. Additionally, TIMP-1-deficient mice experienced a significant reduction in astrogliosis during mouse experimental autoimmune encephalomyelitis (EAE) [95]. EAE involves interactions between immunopathological and neuropathological mechanisms, providing insights into inflammation, demyelination, axonal loss, and gliosis. EAE’s complex neuropharmacology makes it a versatile model for SCIs and other neurological ailments [16,96]. Furthermore, Ogier et al. demonstrated that the treatment of astrocyte cell cultures with TIMP-1 induced greater astrocyte proliferation than those treated with proinflammatory stimuli, such as TNF-α and anti-Fas antibody. Astrocytes present in TIMP-1-deficient mice displayed a decreased response to anti-Fas antibodies compared to their wild-type (WT) counterparts. Therefore, TIMP-1 likely induces astrocyte proliferation to a greater extent than TNF-α and anti-Fas antibodies [97]. Taken together, these findings highlight TIMP-1’s crucial role in modulating astrocytic responses and suggest potential avenues for future investigations.

However, it is important to consider the duration of immune activation when investigating the beneficial effects of TIMP-1 on astrocytes, as there are differences in TIMP-1’s presence during acute and chronic inflammation. During acute immune activation around neuronal lesions, astrocytes elicit an augmentation of TIMP-1 concentrations as a repair response to myelin inflammation [16,93,98,99,100]. These reactive astrocytes act as the primary source of TIMP-1 production in pre-scaring neuronal lesions [101]. Conversely, in cases of chronic activation of TIMP-1, such as in end-stage neurological disease patients, TIMP-1-mediated responses are reduced, with the downregulation of TIMP-1 and discontinuation of TIMP-1-mediated neuroprotection. Chronic inflammation causes the TIMP-1 levels in the CNS to decrease below the homeostatic levels [100]. These findings suggest a difference in astrocytic proliferation between acute and chronic activation. The exact mechanism behind the TIMP-1 activation of astrocytes is largely uncharted; understanding of the duality of TIMP-1 and astrocytic activation may lead to insights for novel treatments of neurological diseases and injuries [92].

#### 2.5.2. TIMP-1’s Effect on Microglia and Macrophages

Similar to its expression in reactive astrocytes, TIMP-1 is also expressed in microglia in response to inflammation and injury. The delivery of TIMP-1 prevents the release of TNF-α from microglia, highlighting its anti-inflammatory and pain-reducing properties. This suggests that TIMP-1 plays a pivotal role in the signaling cascade responsible for reducing inflammation and modulating microglia activation [90]. A study by Crocker et al. demonstrated this concept in TIMP-1-deficient mice with EAE. The TIMP-1-deficient mice exhibited severe myelin pathology compared to WT mice. The disruption of myelin was accompanied by lymphocyte infiltration and macrophage/microglia buildup in the brain parenchyma. Thus, astrocyte expression of TIMP-1 during EAE in WT mice illustrates an intrinsic cytoprotective response through the regulation of microglial activation and macrophage infiltration to the site of injury [10].

#### 2.5.3. TIMP-1’s Effect on Neurons

TIMP-1 is involved in the recovery of sensorimotor function and maintenance of the blood–brain barrier (BBB) [13,16]. Its neuroprotective mechanism is demonstrated after excitotoxic stress, where neuronal cells are exposed to glutamate, causing an over-influx of calcium into the cell, leading to cell death. Tan et al. demonstrated that the introduction of TIMP-1 to neurons via adenovirus-mediated gene transfer provided neuroprotection against excitotoxicity in the CNS by reducing the calcium influx. The addition of a broad MMP inhibitor in hippocampal cultures resulted in no significant difference in glutamate toxicity protection. The inability of broad-spectrum MMP inhibitors to mimic TIMP-1’s effects suggests that the complexes formed by TIMP-1 and MMPs have a differential inhibitory effect, resulting in neuroprotection [102]. Furthermore, cortical neuronal cultures exposed to decreased oxygen, then subsequently oxygenated with the addition of recombinant TIMP-1 in vitro, led to increased neuronal survival. In vivo, TIMP-1 overexpression reduces lesion sizes, MMP-9 expression, and BBB permeability in transgenic TIMP-1 mice post-brain injury [103].

**Figure 4:**
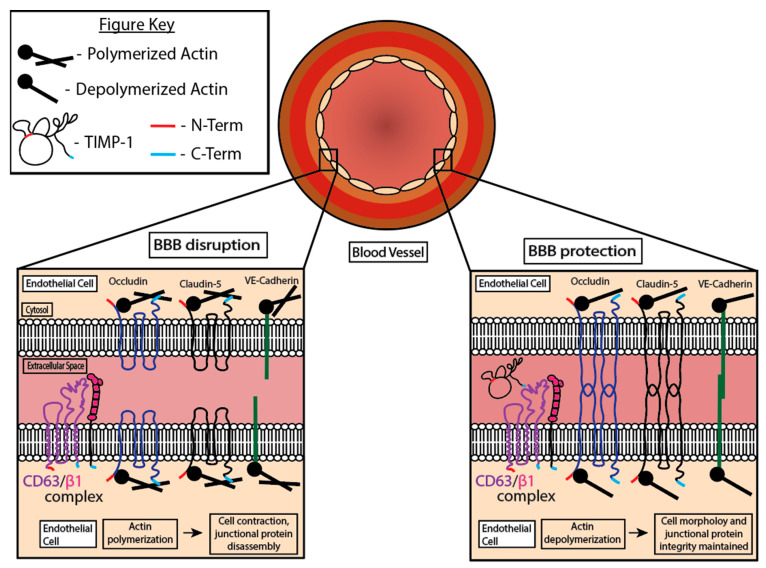
TIMP-1 involvement in the protection of the BBB. TIMP-1 binding CD63/beta1 integrin in blood vessels can allow for proteins in the junction to remain intact. Junction proteins in the BBB hold endothelial cells together and stabilize them. CNS inflammation disrupts the integrity of the junctions in the vessel. This figure is based on the graphical abstract from Tang et al. [91].

The underlying molecular mechanisms of TIMP-1’s neuroprotective effects are not well known. Tang et al. reported that TIMP-1 performs a protective role through amelioration of the BBB breakdown in a mouse model. This mechanistically occurs via TIMP-1 regulation of the endothelial barrier integrity through its interaction with the CD63/integrin β1 complex on the cell surface (Figure 4). The signaling pathway triggered by this process protects against endothelial barrier disruption and stabilizes the actin cytoskeleton, resulting in neuroprotection [91]. This conclusion provides insights into the potential therapeutic significance of TIMP-1’s neuroprotective functions.

#### 2.5.4. TIMP-1’s Effect on Neural Stem Cells

TIMP-1 also affects the localization and differentiation of neural stem cells by acting as a chemoattractant to neural stem cells in brain glioma through CD63 binding [104]. In addition, adding rmTIMP-1 to OPCs for two days yielded enhanced differentiation and increased the number of NG2-positive OPCs, which is crucial in CNS development [16]. In OPCs, which carry out axon myelination, TIMP-1 promotes β-catenin upregulation via Akt, indicating that TIMP-1 has trophic effects in CNS myelination [80].

#### 2.5.5. TIMP-1’s Role in Neuroregeneration and Neuroplasticity

It is speculated that TIMP-1 has a role in CNS regeneration and plasticity [105]. Liu et. al. demonstrated that elevated TIMP-1 synthesis is correlated with sensory axon regeneration in the CNS after a sciatic nerve injury [13]. In addition, TIMP-1 KO mice with EAE experienced reduced myelin regeneration [10]. It was also determined that induced CNS epileptic TIMP-1 KO mice experienced less synaptic remodeling [105]. Using TIMP-1 after a CNS lesion may offer a novel therapy to promote myelination [16]. Thus, TIMP-1 plays a critical role in neuroregeneration, as well as neuroprotection after a CNS injury.

### 2.6. TIMP-1 after a SCI

Although TIMP-1 expression is relatively low in the healthy CNS, it is significantly upregulated post-SCI [16,17,106]. Studies analyzing the gene transcription levels of 100 ECM proteins in rats found that TIMP-1 was the highest upregulated gene one day post-SCI, with the upregulation continuing for 45 days after injury [18,107]. Additionally, studies analyzing the protein levels of 28 cytokines and chemokines involved in inflammation in rats and mice observed that TIMP-1 was upregulated more than any other cytokine tested, peaking 24 h post-SCI and remaining upregulated for at least 7 days post-SCI [17,66]. Astrocytes are the primary cells secreting TIMP-1 in spinal cord regions immediately surrounding the lesion site [16,17,18,106,108,109]. When female rats underwent a SCI, there was a dramatic increase in TIMP-1 expression at the injury epicenter, as well as rostral and caudal to the epicenter post-SCI [110]. Similarly, there is an increased expression of TIMP-1 via astrocytes post-CNS injury (neuroinflammation and stroke) in a mouse model [111]. Moreover, astrocytes cultured in vitro with IL-1β increased TIMP-1 secretion, demonstrating that the activation of astrocytes causes them to upregulate TIMP-1 [100].

Post-mortem samples taken from human patients post-SCI contrast the results found in many rodent studies, as TIMPs were not strongly expressed in the lesion epicenter in the time period of 2–24 days following a SCI [112]. However, an important caveat is the analysis of human tissue was only performed in the lesion epicenter, and it is likely that astrocytes surrounding the lesion expressed TIMP-1.

Investigators using EAE models to understand complex neurological ailments also observed a significant upregulation in TIMP-1 [93,95,96]. In Theiler murine encephalomyelitis and glial fibrillary acidic protein-transgenic mice, there was an increased expression of TIMP-1 in astrocytes and MMPs in the injured and inflamed areas of the CNS. In addition, the inflamed regions with mononuclear cell recruitment showed astrocytes with upregulated TIMP-1 [93]. In a model of EAE with TIMP-1 KO mice, Crocker et al. observed increased numbers of infiltrating immune cells during the acute phase of EAE in TIMP-1 KO mice and less improvement in myelination in the chronic phase compared to wild-type mice with EAE [10]. These EAE results demonstrate the role TIMP-1 has in scar formation after injury.

TIMP-1 directly influences how astrocytes respond to IL-1β. Johnson et al. demonstrated that, after a scratch wound in the primary astrocyte cell cultures, TIMP-1 was upregulated from 4 to 24 h, which led to a robust healing response. However, TIMP-1 KO astrocyte cell cultures only had a minimal healing response to a scratch wound, though the response was improved with IL-1β treatment [85]. The contrasting results in the TIMP-1 KO astrocytes demonstrate the importance of TIMP-1 in controlling astrocyte function.

## 3. Conclusions

The purpose of this review is to provide an in-depth analysis of the role of TIMP-1 after a SCI and its relationship with astrocytes, glial cells, and the ECM. There is extensive literature supporting the role of TIMP-1 in reducing the inflammation and destruction of the ECM through the inhibition of MMP activity. Additionally, TIMP-1 is significantly upregulated post-SCI and primarily operates via two distinct pathways that benefit repair mechanisms: MMP-dependent and MMP-independent pathways. In a neuroprotective manner, TIMP-1 significantly contributes to both ECM remodeling and preservation of the BSCB after a SCI. Although much is known about the role of TIMP-1, the molecular mechanisms behind these functions remain unclear. Further research in this subject would improve our understanding of TIMP-1’s role after a SCI and give insight into novel therapeutic strategies to promote neural repair and functional recovery after a SCI.

## Data Availability

There is no additional data available.
